# PRDX6 knockout restrains the malignant progression of intrahepatic cholangiocarcinoma

**DOI:** 10.1007/s12032-022-01822-9

**Published:** 2022-10-08

**Authors:** Hong Li, Zhengsheng Wu, Rulei Zhong, Qikun Zhang, Qixin Chen, Yuxian Shen

**Affiliations:** 1 School of Basic Medical Sciences, Anhui Medical University, 81 Meishan Road, Hefei, 230032 China; 2 Biopharmaceutical Institute, Anhui Medical University, 81 Meishan Road, Hefei, 230032 China; 3 First Affiliated Hospital of Anhui Medical University, 218 Jixi Road, Hefei, 230032 China

**Keywords:** Intrahepatic cholangiocarcinoma, PRDX6, Wnt7a/b, Mmp7, Ccnd2

## Abstract

Intrahepatic cholangiocarcinoma (ICC) has a poor prognosis. The bifunctional protein peroxiredoxin 6 (PRDX6), which has both calcium-independent phospholipase A2 (iPLA2) and glutathione peroxidase (GPx) activity, participates in the development of multiple tumors. However, the function and clinical significance of PRDX6 in ICC remain unclear. In this study, we characterized PRDX6 in both human ICC and thioacetamide (TAA)-induced rat ICC. We found PRDX6 was significantly increased in ICC tissues, compared with the peritumoral tissues, and PRDX6 expression level was positively correlated with the malignant phenotype in ICC patients. Furthermore, PRDX6 genetic knockout significantly inhibited the tumor progression in rats. By using RNA sequencing analysis, we found 127 upregulated genes and 321 downregulated genes after PRDX6 knockout. In addition, we noticed a significant repression in the Wnt7a/b cascade, which has been shown to play an important role in the occurrence of ICC. We confirmed that gene expressions in the Wnt7a/b cascade were inhibited in ICC tissues after PRDX6 knockout by using qRT-PCR and immunohistochemistry analysis. Collectively, our findings suggest that PRDX6 may promote ICC by regulating the Wnt7a/b pathway, which could be a novel therapeutic target for ICC.

**Supplementary Information:**

The online version contains supplementary material available at 10.1007/s12032-022-01822-9.

## Introduction

Intrahepatic cholangiocarcinoma (ICC) is the second most common malignant tumor in the liver with an increasing incidence worldwide. It has a poor prognosis, accounting for 13% of global cancer mortality [1]. Complete surgical resection is the only treatment option, but few patients (15%) have operable disease [1]. Currently, research on ICC is very limited, and a better understanding of disease biology and new therapies is urgently needed.

The protein PRDX6 is a thiol-containing peroxidase belonging to the peroxiredoxin (PRDX) family with the ability to reduce hydrogen peroxide (H_2_O_2_) [2]. Peroxiredoxins are classified as 1-Cys or 2-Cys PRDXs depending on whether they contain one or two conserved cysteine residues [3]. The protein PRDX6 is a 1-Cys that induces distinct catalytic cycles and uses glutathione (GSH) instead of thioredoxin as a physiologically reducing agent. In addition, PRDX6 is a bifunctional enzyme with phospholipase A2 (PLA2) and peroxidase activities [4]. PRDX6 is upregulated in various human cancers such as esophageal squamous cell carcinoma, colorectal cancer, ovarian cancer, lung cancer, skin cancer and cervical cancer [5–11]. High PRDX6 levels promote cancer progression, migration, invasiveness and resistance to radiotherapy and chemotherapy [5–11]. Both PRDX6 peroxidase and PLA2 activity are involved in tumor development. In lung cancer, PRDX6 promotes tumor development by regulating redox-sensitive pathways such as JAK/STAT, MAPK, AP-1 and JNK [12–14]. However, there are few reports on the expression and function of PRDX6 in ICC. In this study, we aimed to explore the role of PRDX6 in ICC. Therefore, we collected human ICC and thioacetamide (TAA) -induced rat ICC tissue samples to study PRDX6 expression. The role of PRDX6 in ICC was investigated by PRDX6 knockout rats and RNA sequencing.

## Materials and methods

### Human tissue

Specimens from 74 ICC tumors and paired peritumoral tissues were collected from 2009 to 2015 at the First Affiliated Hospital of Anhui Medical University for immunohistochemical analysis. None of the patients received preoperative chemotherapy, radiotherapy, or any other medical intervention. Clinicopathological characteristics of patients were retrieved from medical records and summarized in Table 1. This study was approved by the ethics committee of Anhui Medical University. All patients signed informed consent to use their organizations for scientific research. All diagnoses were based on pathological evidence, and the histological characteristics of the samples were assessed by at least two senior pathologists using the World Health Organization (WHO) classification criteria.

**Table 1:** PRDX6 expression and clinicopathological parameters of the 74 Intrahepatic cholangiocarcinoma patients

Variables	Cases (*n* = 74)	PRDX6 expression, *n* (%)		× 2	*P*
		Low *n* (%)	High *n* (%)		
Age (years)					
< 60	42	13(31)	29(69)	2.53	0.112
≥ 60	32	4(12.5)	28(87.5)		
Sex					
Male	44	9(20.5)	35(79.5)	0.389	0.533
Female	30	8(26.7)	22(73.3)		
Histological type					
Well, moderately	43	14(32.6)	29(67.4)	4.115	**0.043**
Poor	31	3(9.7)	28(90.3)		
Tumor size					
≥ 5 cm	62	14(22.6)	48(77.4)	0	1
< 5 cm	12	3(25)	9(75)		
Tumor number					
Solitary	53	16(30.2)	37(69.8)	5.5	**0.019**
Multiple	21	1(4.8)	20(95.2)		
Lymph node metastasis					
Absent	49	13(26.5)	36(73.5)	0.528	0.468
Present	25	4(16)	21(84)		
Vascular invasion					
Absent	57	13(22.8)	44(77.2)	0	1
Present	17	4(23.5)	13(76.5)		
Perineural invasion					
Absent	63	14(22.2)	49(77.8)	0.135	0.713
Present	11	3(27.3)	8(72.7)		
Distant metastasis					
Absent	61	15(24.6)	46(75.4)	0.125	0.742
Present	13	2(15.4)	11(84.6)		
AJCC TNM stages					
I–II	24	13(33.3)	11(66.7)	17.01	**0.000**
III–IV	50	4(8)	46(92)		

### Immunohistochemistry and immunofluorescence

Immunohistochemistry and immunofluorescence were performed on formalin-fixed paraffin-embedded tissue sections. Standard laboratory methods were used for tissue processing, embedding, sectioning and staining [8, 9]. Antibodies are listed in Supplementary Table 1. Staining intensity and percentage of positive cells were analyzed. Staining intensity was assessed using a standard scale, where: 0 = no yellow; 1 = light yellow; 2 = light brown; 3 = yellowish brown. Percentage of positive cells was assessed by a grading standard, where: 0 = 0–10% positive; 1 = 10–25% positive; 2 = 26–50% positive; 3 = 51–75% positive; 4 = 76–100% positive. The final score was the staining intensity fraction multiplied by the positive cell fraction. 0 points were considered negative (−); 1–4 points were considered weak positive (+); 5–8 points were considered positive (+ +); 9–12 points were considered strong positive (+ + +). Other samples with brown-yellow staining were considered positive. For the result of immunofluorescence, images of the BX53 microscope (Olympus, Japan) were used.

### Animal and thioacetamide-induced ICC model

PRDX6 knockout rats were obtained by gene targeting from the Institute of Model Animals of Nanjing University. SD rats as wild-type control group were purchased from Beijing Weitong Lihua Laboratory Animal Technology Co., Ltd. They were reared in the Laboratory Animal Center of the School of Life Sciences, University of Science and Technology of China. Prior to the experiment, each rat was identified as homozygous. Female rats aged 7–8 weeks were fed according to the TAA-induced ICC model. The rats were given water containing 300 mg /L TAA, their health status was recorded. After 27 weeks, the rats were weighed and then anesthetized with chloral hydrate. Liver and other organs were weighed, photographed, fixed and frozen. Animal studies were performed in accordance with the procedural guidelines of Anhui Medical University and were approved by animal welfare and ethical approval agencies.

### Liver tumor analysis

The number of tumors in each liver lobe was observed and counted. Routine hematoxylin–eosin (HE) staining was used to assess signs of malignancy, and Image J (NIH) was used to quantify tumor area as a proportion of liver area in a blinded trial.

### Western blotting

Liver tissues were lysed in lysis buffer and supernatants were collected after centrifugation (12000 rpm, 4 °C for 20 min). 13.5% SDS-PAGE with equal amounts of tissue proteins followed by western blotting against corresponding antibodies. Supplementary Table 1 is the primary antibodies used in these assays. Proteins were detected using a gel trap 3400 (Shanghai, China) and data were analyzed using a gel analyzer in the clinx file system (Shanghai, China). GAPDH was used as a protein loading control.

### RNA sequencing

RNA sequencing analysis was performed on three rats in each experimental group to detect changes in gene expression in tumor tissues of wild-type and PRDX6 knockout groups. The rapidly frozen liver tissue was thawed at 4 °C overnight, and tumor tissue samples were obtained by an anatomical microscope. The tumor tissue was then transported on dry ice to Shanghai Maibo Biomedical Technology Co., Ltd. (Shanghai, China) for subsequent RNA extraction and sequencing. Pearson correlation cluster thermograms were generated using the R software package. The multiple variation and false detection rates for differential gene expression by gene-set analysis (GSA) were 1.5 and 0.5, respectively. The significant gene changes emphasized by GSA were introduced into Gene Unified Pathway analysis for gene-set enrichment analysis.

### qRT-PCR

Liver tissue was frozen in liquid nitrogen and stored at − 80 °C. RNA was extracted by Trizol (Invitrogen, USA) using standard protocols and complementary DNA was generated from 1 mg RNA using a reverse transcriptase kit (Takara, Japan). RT-PCR was performed using an ABI Prism 7000 (Applied Biosystems of Life Technologies, Carlsbad, CA, USA) and SYBR Green reagents (Toyota, Japan). The reaction was divided into two steps and gene expression was standardized by the GAPDH value. Primer sequences were available upon request. Results were obtained as the threshold cycle (CT) values. Expression levels were calculated using the 2-ΔΔCT method.

### Statistical analyses

SPSS (V24.0) was used for statistical analysis, and the data was expressed as mean ± SEM. D'Agostino and Pearson normality tests were used to assess data distribution, and Student’s *t* test or Mann–Whitney *U* test was used to compare the two groups. Clinical data was analyzed by Chi-square test or corrected Chi-square test.

## Results

### PRDX6 is highly expressed in human ICC tissues and correlates with tumor progression

**Fig. 1:**
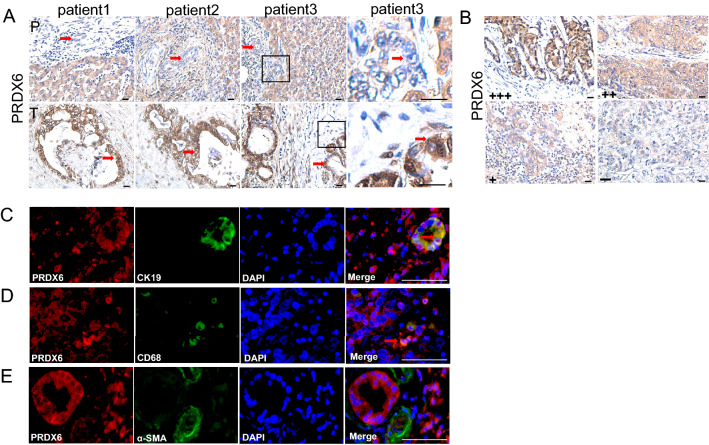
PRDX6 is highly expressed in human ICC tissues and is associated with tumor progression. **A** ICC tissue sections were stained with anti-PRDX6 antibody by Immunohistochemistry. Representative images of PRDX6 tissue array samples (P, peritumoral; T, tumor). **B** Immunohistochemistry showing no (−), low (+), moderate (+ +) and strong (+ + +) staining of PRDX6 in ICC tissues. **C**–**E** Double fluorescence of PRDX6 (red) with CK19, CD68 + , αSMA (green) in ICC tissues. (scale bar, 20 μm)

To assess the expression of PRDX6 protein in ICC species, ICC and peritumoral tissue from 74 patients were subjected to immunostaining analysis, which showed elevated PRDX6 expression in the tumor group compared to the peritumoral group (Fig. 1A). PRDX6 is located in the cytoplasm and nucleus (Fig. 1A). The samples were divided into high expression (+ + or + + +) and low expression (− or +) groups (Fig. 1B). Statistical analysis was performed to determine the relationship between PRDX6 expression level and age, sex, histological type, tumor size, tumor number, lymph node metastasis, vascular invasion, perineural invasion, distant metastasis and TNM stage. Statistical results of PRDX6 expression were associated with histological type (*P* = 0.043), tumor number (*P* = 0.014) and TNM stage (*P* = 0.015), but not with age (*P* = 0.112), gender (*P* = 0.533), tumor size (*P* = 1), lymph node metastasis (*P* = 0.468), vascular invasion (*P* = 1), perineural invasion (*P* = 1) and distant metastasis (*P* = 0.742) (Table 1). Immunofluorescence showed that PRDX6 was expressed in cancer cells (Fig. 1C) and ICC macrophages (Fig. 1D), but not in hepatic stellate cells (Fig. 1E).

### PRDX6 is highly expressed in rat ICC tissues

**Fig. 2:**
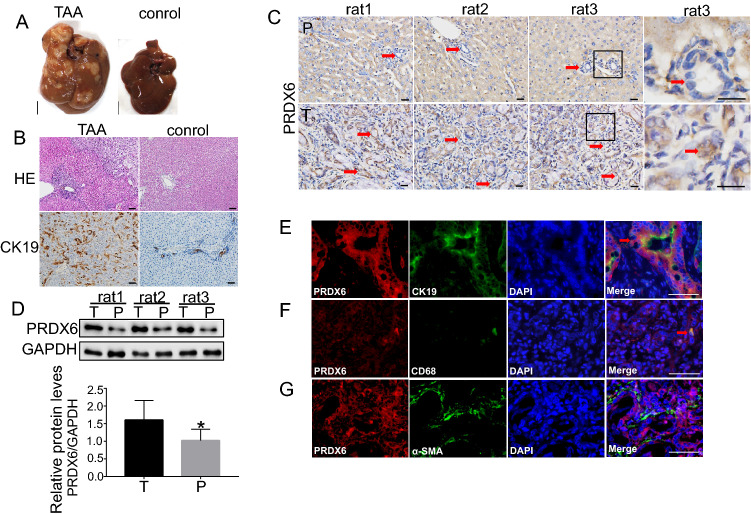
PRDX6 is highly expressed in rat ICC tissues. **A** Liver photographs of female rats (*n* = 3) treated with 300 mg/L TAA for 27 weeks and control (scale bar, 1 cm). **B** Representative HE and CK19-stained liver sections of rat ICC tissues and control (Scale bar, 100 μm). **C** PRDX6 immunostained liver sections from rat ICC tissues (scale bar, 20 μm). **D** PRDX6 was measured in rat ICC tumor and peritumoral tissues by western blotting. GAPDH was used as loading control. **E**–**G** Double fluorescence of PRDX6 (red) in rat ICC tissues with CK19, CD68 + , αSMA (green) (scale bar, 20 μm). (*P* peritumoral, *T* tumor**)**

To investigate the effect of PRDX6 on ICC, we used a rat model induced by TAA, which acts as a hepatoma drug to induce secondary injury in rat hepatocellular carcinoma [15]. Tumors were seen in the liver after rats were given a dose of 300 mg/L TAA in their drinking water for 27 weeks (Fig. 2A). The control group used sterile water instead of TAA. Hepatic tissue HE and the ductal marker CK19 staining showed ICC tumor tissue (Fig. 2B). Immunohistochemical analysis of ICC tumors and peritumoral tissues showed that PRDX6 expression was higher in tumor tissues than in peritumoral tissues (Fig. 2C). PRDX6 was distributed in the cytoplasm and nucleus (Fig. 2C). Western blotting showed that the expression of PRDX6 in rat ICC tumor was higher than that in peritumoral tissue (Fig. 2D). Immunofluorescence showed that PRDX6 was expressed in cancer cells (Fig. 2E) and macrophages (Fig. 2F) in ICC, but not in hepatic stellate cells (Fig. 2G). The above results indicated that the expression of PRDX6 in the rat ICC model was consistent with that of human ICC.

### PRDX6 knockout restrains ICC progression in rats

**Fig. 3:**
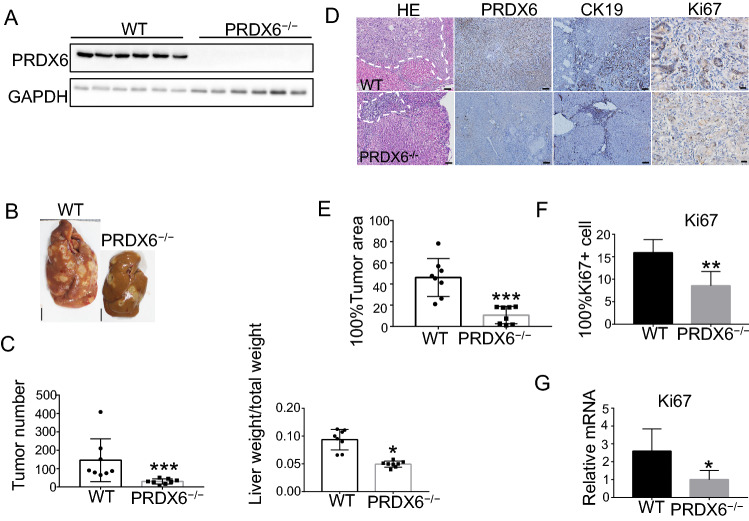
Genetic deletion of PRDX6 inhibits ICC progression in rats. **A** Representative pictures of western blots specific for PRDX6 protein and GAPDH loading control. **B** Liver tiling and low magnification photomicrographs of wild-type (*n* = 8) and PRDX6 knockout female rats (*n* = 8) after 27 weeks of TAA (Scale bar, 1 cm). **C** Tumor number and liver weight/total weight in two groups of rats (*n* = 8). **D** Representative sections stained for HE, PRDX6, CK19 (scale bar, 100 μm) and Ki67 (scale bar, 20 μm) from two groups of rats. Dotted line, tumor border. **E** The total area of the slice and the area of the tumor were measured. Tumor area was calculated as a percentage of the total area. Ten fields of view were taken for each sample and averaged (*n* = 8). **F** Ki67 positive cells were quantified. Ten fields of view were taken for each sample to count the proportion of positive cells and averaged (*n* = 8). **G** qRT-PCR of Ki67 expression in tumor tissues of two groups (*n* = 3). Results are presented as mean ± SEM; Student's *t* test, *P < 0.05, **P < 0.01, ***P < 0.001. (wild-type *WT*, PRDX6 knockout *PRDX6.*^*−/−*^)

The PRDX6 knockout condition was verified by Western blot (Fig. 3A). Well-differentiated ICCs were observed in wild-type and PRDX6 knockout female rats 27 weeks after TAA modeling (Fig. 3B). We calculated tumor number and liver weight/total weight. The mean number of tumors was reduced in the PRDX6 knockout group compared to the wild-type group [157.143 ± 121.136 in wild-type rats (*n* = 8) vs. 30.375 ± 13.773 in PRDX6 knockout rats (*n* = 8)] (*P* < 0.001). And liver weight/total weight decreased [0.0935 ± 0.0185 in wild-type rats (*n* = 8) vs. 0.0495 ± 0.0055 in PRDX6 knockout rats (*n* = 8)] (*P* < 0.05). These differences were statistically significant (Fig. 3C). Microscopic foci of invasive ICC were evident in all groups (Fig. 3D). Immunohistochemical analysis showed that the expression of PRDX6 was low in the knockout group (Fig. 3D). The ductal marker CK19 was positive in both groups of tumor tissue (Fig. 3D). We measured the total area of the sections and tumors separately. Tumor area was calculated as a percentage of the total area. We took 10 fields of view from each sample and calculated the average. Mean tumor area/total area was reduced in the PRDX6 knockout group compared to the wild-type group [46.169 ± 17.873 in wild-type rats (*n* = 8) vs. 10.651 ± 8.223 in PRDX6 knockout rats (*n* = 8)] (*P* < 0.001) (Fig. 3E). Ten fields of view were taken for each sample to count the proportion of positive cells and averaged. Ki67 positive cells in the tumor tissue of the knockout group were significantly reduced (*P* < 0.01) (Fig. 3F). In the qRT-PCR assay, the expression of Ki67 in the knockout group was lower than that in the wild-type group (*P* < 0.05) (Fig. 3G). In conclusion, knockout of PRDX6 restrains ICC progression in rats.

### RNA sequencing analysis of ICC tumor tissue in wild-type and PRDX6 knockout rats

**Fig. 4:**
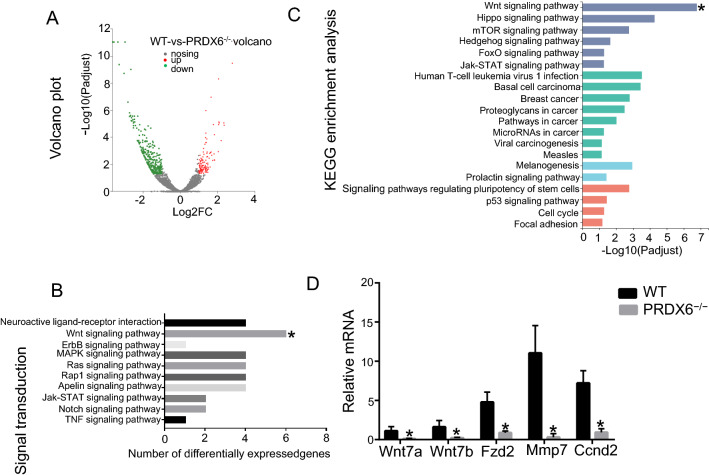
RNA sequencing analysis of livers tumor tissue in wild**-**type and PRDX6 knockout rats. **A** Volcano plot of differentially expressed genes (DEGs) between the two groups. Red dots represent upregulated genes in ICC (*n* = 127), green dots represent downregulated genes in ICC (*n* = 321), and black dots represent genes that were not differentially expressed between the two groups. **B** Top 10 pathways for signal transduction among KEGG pathway events of DEGs between the two groups. **C** KEGG enrichment analysis between the two groups. **D** Changes in Wnt signaling pathway gene expression. (wild-type *WT*, PRDX6 knockout *PRDX6.*^*−/−*^)

To explore the mechanism of action of PRDX6 in ICC, we performed RNA sequencing of tumor tissue from the livers of wild-type and PRDX6 knockout female rats 27 weeks after TAA modeling (*n* = 3). RNA sequencing data showed that 448 annotated genes were differentially expressed in tumor tissue of wild-type and PRDX6 knockout groups, including 127 upregulated genes and 321 downregulated genes (DEseq, fold change ≥ 1.5 and FDR ≤ 0.05) (Fig. 4A). KEGG pathway analysis showed the greatest changes in signal transduction pathways, and 47 cellular signal transduction pathways were altered (Fig S1), including neuroactive ligand–receptor interaction, Wnt signaling pathway, ErbB signaling pathway, MAPK signaling pathway and Ras signaling pathway. The Wnt signaling pathway is the most varied pathway (Fig. 4B). KEGG enrichment analysis also revealed that the Wnt signaling pathway was the most altered pathway (Fig. 4C), showing that Wnt7a, Wnt7b, Fzd2, Mmp7 and Ccnd2 were downregulated upon PRDX6 knockout (Fig. 4D).

### PRDX6 knockout represses the Wnt7a/b cascade

**Fig. 5:**
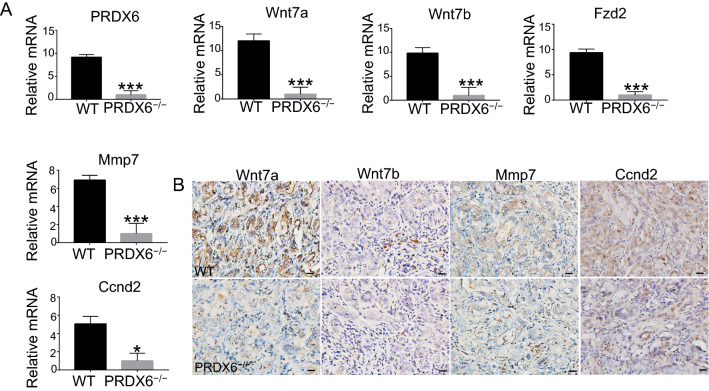
PRDX6 knockout inhibits the Wnt7a/b cascade. **A** RNA extraction from tumor tissue. qRT-PCR results showed the expression of PRDX6, Wnt7a, Wnt7b, Fzd2, Ccnd2 and Mmp7 in the two groups. GAPDH was used as a loading control (*n* = 5). **B** Representative staining for Wnt7a, Wnt7b, Ccnd2, and Mmp7 in the two groups of tumor tissue sections. (scale bar, 20 μm) (wild-type *WT*, PRDX6 knockout *PRDX6.*^*−/−*^)

To further validate our results, ICC tumor tissues were taken from the wild-type and PRDX6 knockout groups, and RNA sequencing results were confirmed by qRT-PCR and immunostaining. qRT-PCR was used to detect the knockout effect of PRDX6 in the model rat tumor tissues (Fig. 5A). qRT-PCR results showed that the expression of Wnt7a, Wnt7b, Fzd2, Mmp7 and Ccnd2 in the PRDX6 knockout group was lower than that in the wild-type group (Fig. 5A). Immunostaining showed lower protein expression of Wnt7a, Wnt7b, Mmp7 and Ccnd2 in tumor tissues of the PRDX6 knockout group compared to the wild-type group (Fig. 5B). The protein was not detected, as there are no rat immunohistochemical antibodies against Fzd2. Wnt7a protein is mainly expressed in tumor cells, and Wnt7b is mainly expressed in macrophages (Fig. 5B). These results suggest that PRDX6 knockdown inhibits the Wnt7a/b cascade.

## Discussion

PRDX6 belongs to the PRDX family, and unlike other members of this family, it has both glutathione peroxidase (GPx) and PLA2 activity. In addition, PRDX6 both promotes and inhibits cancer [16]. However, it is unclear what role PRDX6 plays in ICC. To explore this question, 74 ICC carcinomas and peritumoral tissues were collected for PRDX6 immunohistochemical analysis. We found that the expression of PRDX6 was higher in cancer tissues than in peritumoral tissues. Analysis of PRDX6 protein expression levels combined with clinicopathological data indicated that PRDX6 was associated with tumor differentiation, tumor number and TNM stage. The above suggests that PRDX6 may play a role in ICC. Immunofluorescence analysis showed that PRDX6 was expressed in cancer cells and macrophages, but not in hepatic stellate cells. In the TAA-induced ICC rat model, the expression of PRDX6 was consistent with that of human ICC. Since PRDX6 is expressed in several kinds of cells in ICC, complete knockout allows better study of the function of this gene. The PRDX6 knockout animal technology is relatively mature, and knockout PRDX6 in mice does not affect the survival of animals, nor does it produce tumors [17, 18]. In this study, PRDX6 knockout rats were used to establish an ICC model to study the role of PRDX6 in ICC.

Since there is no gender difference in ICC, female rats aged 7–8 weeks were selected to model, and the experiment was terminated after 27 weeks according to the literature and pre-experiment results. The number of tumors, liver/body weight ratio and HE staining tumor area in the knockout group were less or lower than those in the wild-type group. Knockout of PRDX6 restrains ICC progression in rats. The results suggest that PRDX6 may promote the development of ICC.

PRDX6 is expressed in ICC cancer cells and macrophages, the latter being a key cellular liver component for maintaining liver homeostasis. Studies have shown that macrophages in ICC are closely related to T-cell density, vascular endothelial cell apoptosis and tumor microenvironment, and some macrophages may be indicators for evaluating prognosis and clinical efficacy [19]. This makes it more difficult to study the cancer-promoting mechanism of PRDX6. Based on the above, we chose to sequence the tumor tissue transcriptomes of wild-type and PRDX6 knockout rat ICC models to obtain more comprehensive information.

Sequencing results showed that Wnt7a, Wnt7b, Fzd2, Mmp7 and Ccnd2 in the Wnt signaling pathway were downregulated after PRDX6 knockout, suggesting that this signaling pathway may be involved in the regulation of PRDX6's cancer-promoting effect. The canonical Wnt signaling pathway in mammals is regulated by the transcription factor β-Catenin. APC/Axin/CK1/GSK3β-disruption complex is inhibited when Wnt ligands bind to Frizzled family receptors and common receptors of the lrp-5/6/arrow family, resulting in β-catenin stability and its translocation to the nucleus. This provides a transcriptional activation domain for TCF/lef protein binding that activates target genes for cell proliferation, apoptosis and cell cycle [20]. Aberrant activation of Wnt/β-Catenin signaling was observed in ICC and correlated with the degree of malignancy and patient prognosis. Increased transcription of Wnt ligands, especially Wnt7b, is mainly due to secretion by macrophages in cholangiocarcinoma [21, 22]. The study showed that in ICC patients, the Wnt ligands Wnt7b and Wnt10a were highly expressed in tumor tissues [21]. The Wnt7b protein often co-localizes with the macrophage marker CD68. Transcript levels of β-catenin target proteins such as Ccnd2, CDKN2A, and BIRC5 are known to be increased in tumor patients. Boulter et al. [21] found that the disappearance of macrophages prevented the canonical Wnt signaling cascade (loss of the Wnt7b signal in tumors), resulting in a reduction in tumor number and size. These results suggest that macrophage-secreted Wnt7b plays an important role in the tumorigenesis of ICC. In this study, we found that PRDX6 is expressed in ICC macrophages. In the PRDX6 knockout rat ICC model, the expressions of Wnt7b, Fzd2 and the target gene Ccnd2 were downregulated. These results suggest that PRDX6 may promote ICC by regulating the Wnt7b/Ccnd2 pathway in macrophages.

The total amino acid homology of Wnt7a and Wnt7b was 76.5% [23], and there are significant differences in expression, localization and function between them. Wnt7a has dual roles in human tumors. It is downregulated in lung cancer and exerts anticancer effects [24]. However, it is upregulated in ovarian cancer [25], breast cancer [26], glioma [27] and other malignant tumors, and exerts a cancer-promoting effect. Overexpression of Wnt7a is associated with inflammation and fibrosis [28, 29], but its exact role requires further study. Wnt7a is abundant in ovarian cancer cells and can promote the proliferation, adhesion and invasion of cancer cells in cell experiments, and reduce the tumorigenicity of Wnt7a knockdown cells in vitro. The mechanism is that Wnt7a promotes Mmp7 expression, thereby promoting tumorigenesis [30]. We found that Wnt7a, Fzd2 and Mmp7 were downregulated after PRDX6 knockdown, suggesting that PRDX6 may promote carcinogenesis by regulating the Wnt7a/Mmp7 pathway. qRT-PCR was used to validate the sequencing results. Wnt7a, Wnt7b, Fzd2, Mmp7 and Ccnd2 expression was downregulated after PRDX6 knockdown. In addition to Fzd2 without immunohistochemical antibodies of rat, the protein levels of Wnt7a, Wnt7b, Mmp7 and Ccnd2 were also downregulated in ICC. Wnt7a is mainly expressed in cancer cells and Wnt7b is expressed in macrophages. PRDX6 may promote the occurrence and development of ICC by regulating Wnt7a/Mmp7 in cancer cells and Wnt7b/Ccnd2 in macrophages.

In conclusion, we found that the expression of PRDX6 in ICC was higher than in peritumoral tissues, which was related to the degree of tumor differentiation, tumor number and TNM stage. PRDX6 is expressed in cancer cells and macrophages. After the ICC model was established in PRDX6 knockout rats, malignant progression and Ki67 positive cells in the knockout group were reduced, indicating that PRDX6 knockout affects the progression of ICC. Sequencing showed that the expression of Wnt7a/b, Ccnd2, Mmp7 and Fzd2 was downregulated after PRDX6 knockout. PRDX6 may promote cancer in ICC by regulating this pathway, but the specific regulatory mechanism is unclear. Further research is warranted.

## Supplementary Information

Below is the link to the electronic supplementary material.

Supplementary file1 Supplementary Table 1 lists the antibodies. Supplementary Table 2 lists the primer. Supplementary Table 3 Expression of PRDX6 in ICC tumor and peritumoral tissues. Figure S1 KEGG pathway incident of DEGs between two groups (DOCX 89 KB)
