# Supplementary material for: PRDX6 knockout restrains the malignant progression of intrahepatic cholangiocarcinoma
Source: Med Oncol. 2022 Oct 8;39(12):250. doi: 10.1007/s12032-022-01822-9 (PMC9547796; doi:10.1007/s12032-022-01822-9)

Supplementary Table 1 lists the antibodies

（1）PRDX6 Abcam Ab16947

（2）PRDX6  Abcam Ab59543

（3）CK19 Abcam Ab52625

（4）CD68 Abcam Ab955

（5）α-SMA Abcam Ab5831

（6）GAPDH proteintech 60004-1-Ig

（7）Ki67 Abcam Ab9449

（8）Ki67 proteintech 27309-1-AP

（9）Wnt7a  Affinity [DF7338](http://www.affbiotech.cn/goods-6132-DF7338-Wnt7a_Antibody.html)

（10）Wnt7b Affinity [DF9042](http://www.affbiotech.cn/goods-12515-DF9042-WNT7B_Antibody.html)

（11）Fzd2 Affinity [DF2791](http://www.affbiotech.cn/goods-6872-DF2791-FZD2_Antibody.html)

（12）Ccnd2 Affinity [AF5410](http://www.affbiotech.cn/goods-4716-AF5410-Cyclin_D2_Antibody.html)

（13）Mmp7 Affinity [AF0218](http://www.affbiotech.cn/goods-89-AF0218-MMP7_Antibody.html)

（14）Alexa Fluor 488 goat anti-mouse IgG ( H+L) Invitrogen A11029

（15）Alexa Fluor 568 goat anti-rabbit IgG (H+L) Invitrogen A11036

Supplementary Table 2 lists the primer

| Primer Forword Reverse | | |
| --- | --- | --- |
| GAPDA | CAACTCCCTCAAGATTGTCAGCA | GGCATGGACTGTGGTCATGA |
| PRDX6 | TGACAGCCCGTGTGGTATTC | GTCAGCTGGAGGGAGTCAAC |
| Ki67 | AGGACTTTGTGCTCTGTGACC | CTCTTTTGGCTTCCATTTCTTC |
| Wnt7a | CTCTGCCGACATCCGGTAC | CGACCCGCCTCGTTATTG |
| Wnt7b | AGAAGCAAGGCTACTACTACCA | TGCCTCATTGTTGTGAAGGT |
| Fzd2 | CAGGGCACTTAGAAAGAAGGCT | AGGAACCAGGTGAGGGACAGA |
| Ccnd2 | TCCTGGCCTCCAAACTCAAAG | GAGGCTTGATGGAGTTGTCG |
| Mmp7 | GGCTTTAACCATGTGGGGCA | GGCCCATCAAATGGGTAGGA |

Supplementary Table 3 Expression of PRDX6 in ICC tumor and peritumoral tissues

| Groups Scores（͞x±s） | | Grading of PRDX6 expression | | | | Positive rate |
| --- | --- | --- | --- | --- | --- | --- |
|  |  | **‒** | **＋** | **＋＋** | **＋＋＋** |  |
| T(n=74) | 7.09±2.65 | 4 | 13 | 29 | 28 | 94.6% |
| P(n=74) | 0.8±12.6 | 46 | 28 | 0 | 0 | 38% |

P, peritumor; T, tumor

Figure S1 KEGG pathway incident of DEGs between two groups


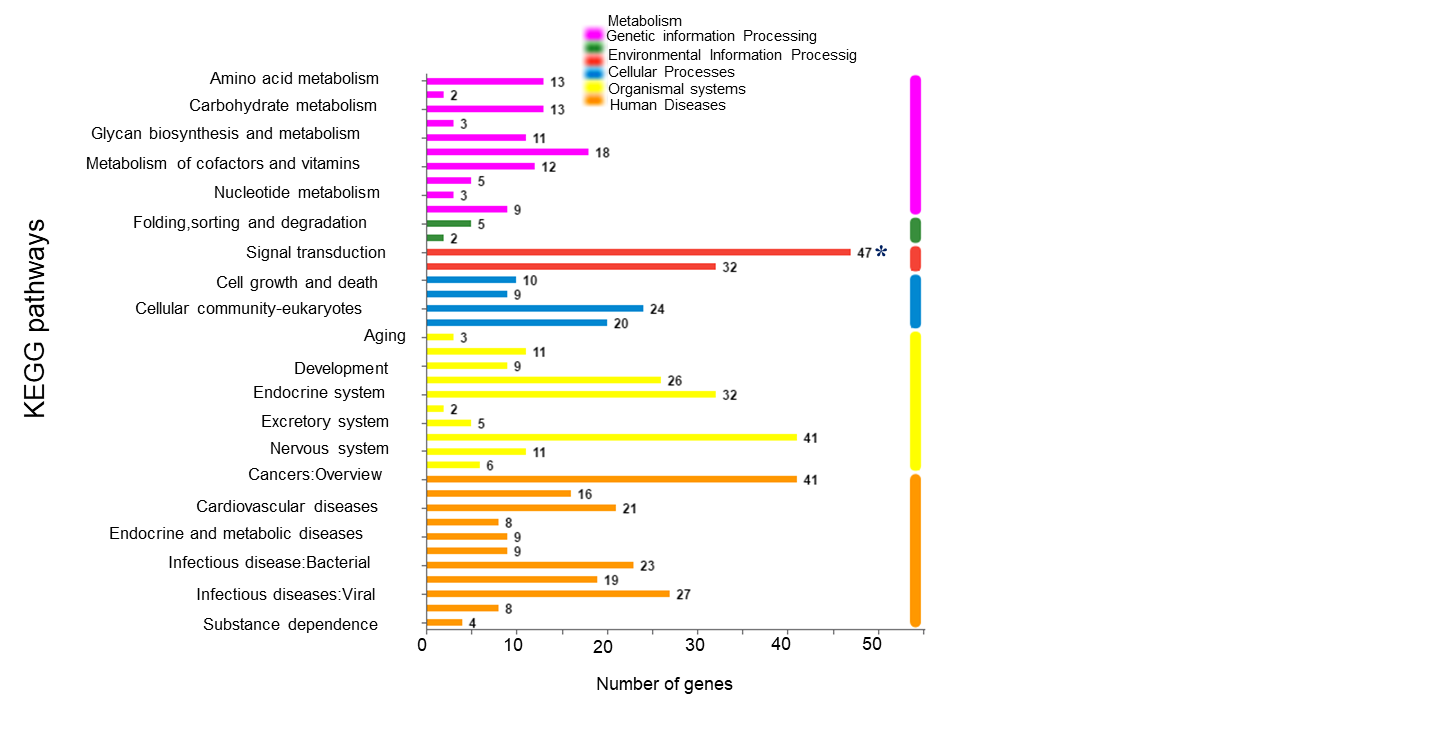

Supplement: Supplementary file 1 — Supplementary file1 Supplementary Table 1 lists the antibodies. Supplementary Table 2 lists the primer. Supplementary Table 3 Expression of PRDX6 in ICC tumor and peritumoral tissues. Figure S1 KEGG pathway incident of DEGs between two groups (DOCX 89 KB) [file 12032_2022_1822_MOESM1_ESM.docx]
